# Competition on the Rocks: Community Growth and Tessellation

**DOI:** 10.1371/journal.pone.0012820

**Published:** 2010-09-30

**Authors:** Espen Jettestuen, Anders Nermoen, Geir Hestmark, Einar Timdal, Joachim Mathiesen

**Affiliations:** 1 Physics of Geological Processes, University of Oslo, Oslo, Norway; 2 International Research Institute of Stavanger, Stavanger, Norway; 3 Department of Biology, University of Oslo, Oslo, Norway; 4 Natural History Museum, University of Oslo, Oslo, Norway; 5 Niels Bohr Institute, University of Copenhagen, Copenhagen, Denmark; University of Milano-Bicocca, Italy

## Abstract

Crustose lichen communities on rocks exhibit fascinating spatial mosaics resembling political maps of nations or municipalities. Although the establishment and development of biological populations are important themes in ecology, our understanding of the formation of such patterns on the rocks is still in its infancy. Here, we present a novel model of the concurrent growth, establishment and interaction of lichens. We introduce an inverse technique based on Monte Carlo simulations to test our model on field samples of lichen communities. We derive an expression for the time needed for a community to cover a surface and predict the historical spatial dynamics of field samples. Lichens are frequently used for dating the time of exposure of rocks in glacial deposits, lake retreats or rock falls. We suggest our method as a way to improve the dating.

## Introduction

Lichens are composite organisms of fungi and algae (mycobionts and photobionts) living in a symbiotic relationship [1] possibly including bacterial microsymbionts [2]. This form of relationship is believed to be as old as 600 million years [3]. The photobiont, commonly a unicellular green alga, is situated in a matrix of fungal hyphae which keep the overall organism (the lichen thallus) coherent and attached to the substrate. Lichens may reproduce by propagules containing the intact symbiosis (e.g. soredia, isidia, thallus fragments) or by asexual fungal conidia or sexually generated fungal spores which need to link up with an alga in nature to re-establish the symbiosis [4]. With the exception of a few parasitic lichen species, lichens do not establish themselves on top of pre-existing lichen individuals. Lichens exhibit many growth forms but one major group dominating rock surface habitats are the crustose lichens, thalli closely attached to the substrate, growing radially outwards from the point of establishment (nucleation). Crustose lichens have indeterminate growth, i.e. they grow until they meet other lichens or some other kind of environmental obstacle. When a crustose lichen meets another, a contact boundary is formed, and if the lichens are competitively equal these boundaries may remain stable over time. The slow growth of crustose lichens have however so far been a major obstacle to obtaining data on the temporal dynamics of such borders and possible intra- or interspecific competitive hierarchies.

**Figure 1:**
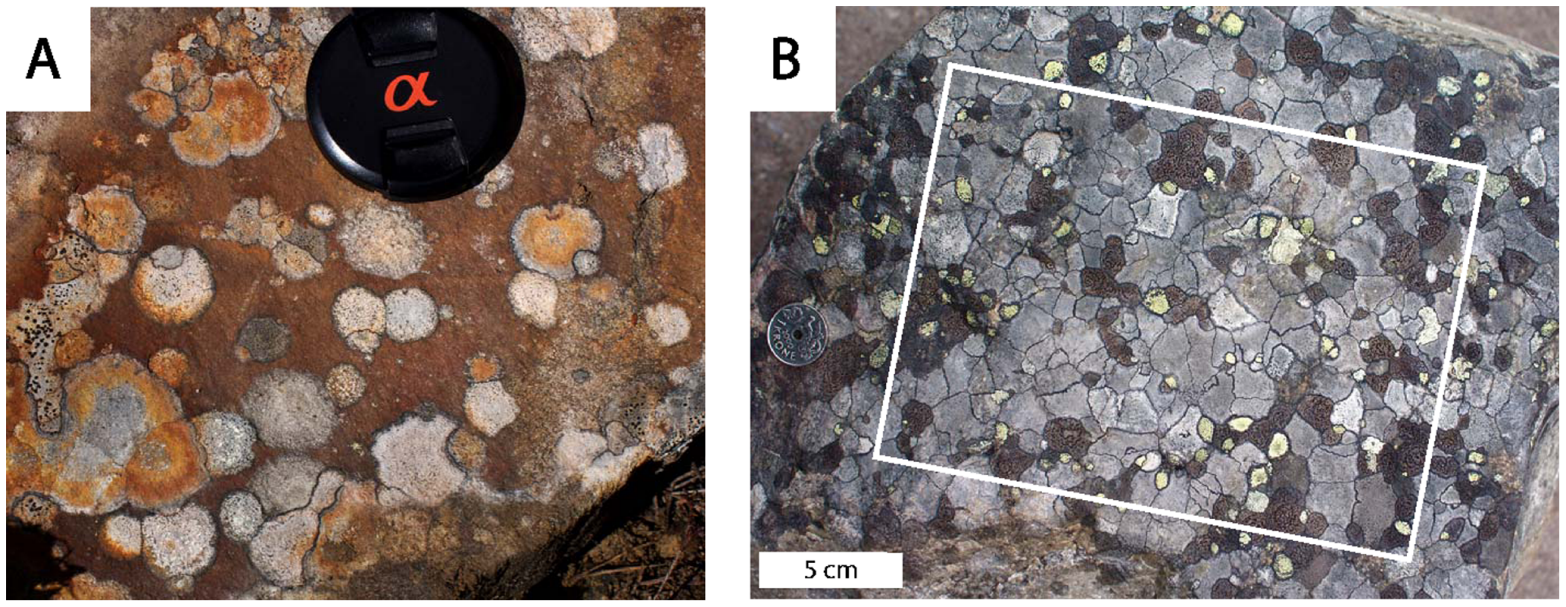
Photographs of lichen communities. A) Photograph of a partially covered rock which has been exposed for approximately 60 years (when a nearby road was built). The diameter of the lens cap is approximately 6 cm. B) Picture of a rock completely covered by lichens. Both rocks have been located in an open pine forest at 700 m altitude in Oppland, Norway.

The collective growth of adjacent crustose lichens form a complex pattern with similarities to the patterns formed by Voronoi tessellations of space, see Fig. 1, and this model has been used to analyze interactions in some foliose lichens where thalli can overgrow each other and effectively compete for light [5], [6]. In crustose lichens the map pattern formed is the result of a dynamical process where individual species compete for unoccupied surface to establish themselves, grow and reproduce. Here we propose a model of the full dynamics of lichen communities and test it on numerical simulations and field samples of lichens.

## Results

### Growth model

**Figure 2:**
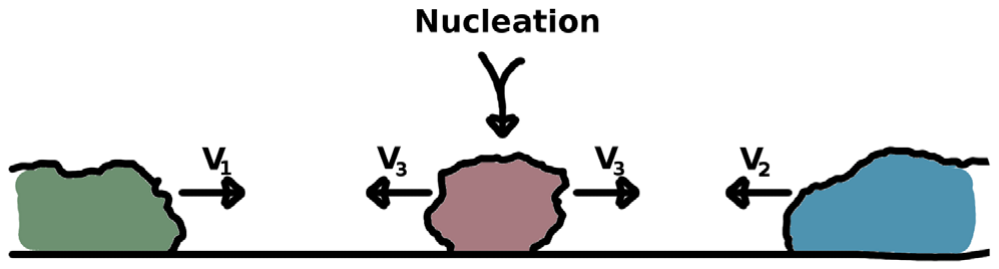
Model of lichen growth and establishment. New lichens are nucleated in the space between existing lichens at a rate 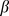. The ongoing nucleation greatly accelerates the overall rate at which the unoccupied space is being covered.

On sufficiently smooth surfaces, lichens grow radially from their points of establishment and may after some possible transient for small radii [7], [8] be assumed to have radii proportional to their age. This is consistent with population growth and spreading models such as Fisher's equation where, although the dynamics might be rather complicated [9], populations spread with characteristic velocities. During the growth of existing lichens, new individuals nucleate in the uncovered space from diaspores dispersed by nearby or remote lichens and where lichens meet, networks of contact boundaries start to form (marked by black lines in Fig. 1B). Our model is formulated in terms of the competition for unoccupied space and an expression will be derived for the time it takes to reach full coverage. For that purpose, we first consider the dynamics of lichens located on a long strip, i.e. the growth is confined to one dimension. The uncovered space between two lichens with growth velocities 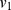 and 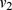 will contract at an overall velocity 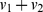. However, as seen in Fig. 2, the contraction is further accelerated by the introduction of a new lichen with growth velocity 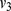 in the gap, that is the gap is divided in two regions which again will contract with the new velocities 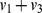 and 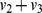. We now consider the simultaneous evolution of many such gaps on the long strip. The average number of gaps of size 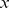 per unit length at a time 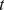 is given by the function 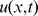. We assume that lichens nucleate in the unoccupied space at a rate 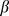 (in units of 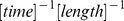) and grow approximately by an average velocity 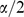 (in units of 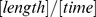), i.e. our model contains two parameters only. The simplification of using the same velocity for all lichens is justified as long as the intra-species variability is sufficiently small. We then arrive at the following integro-differential equation for the evolution in time of 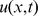,
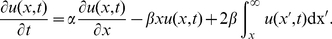
(1)


The first term on the right hand side describes the continuous shrinking of gaps, the second term describes the number of gaps of size 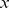 which are divided by the nucleation of new lichens and the last term describes the number of gaps of size 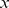 produced from the division of gaps of size 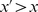. In general, a gap of size 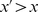 can by the nucleation of a lichen provide a gap of size 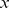 in two ways which explains the factor 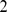 in the equation. Population balance equations similar to Eq. (1) have been used to describe a wide range of phenomena, including the dynamics of microtubules [10], size distribution of 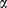 helices [11] and grain growth [12]. Starting with one big gap of size 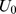, Eq. (1) has a solution on the form
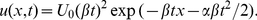
(2)


Here we are interested in knowing the time it takes to cover fully an area, which is the same as knowing the time it takes for the unoccupied area to vanish. The total gap size 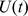 follows from an integration of the distribution over all gap sizes 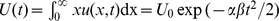.

From this equation we find that a given coverage 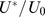 (in percent of initial gap size) of the strip is reached at a time 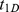 given by the expression
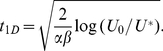
(3)From Eq. (2) we also find an expression for the average size 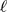 of a lichen, which is the length of the system divided by the total number of lichens introduced. The number of lichens follows from an integration over time of 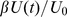 and the reciprocal of this integral is the average size, 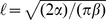.

**Figure 3:**
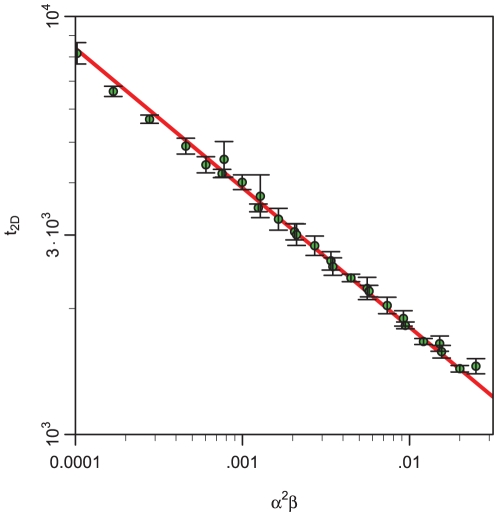
Scaling of the time needed to cover an area. The characteristic time 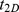 (in arbitrary units) is shown on double logarithmic axes and as function of 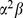. The points and corresponding error bars are computed using the particle production model outlined in the Materials and Methods section. The straight line is the behavior predicted from the model Eq. (4).

**Figure 4:**
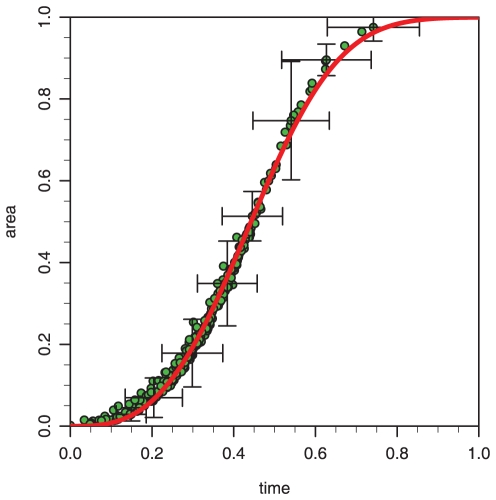
Evolution of the area covered by a lichen community. We compare the predicted evolution of covered area in the field sample shown in Fig. 1A with the model Eq. (5) marked by the line. Each point represents the total area covered at the time of nucleation of a new lichen and is computed from the Monte Carlo simulations. The points are averaged over 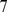 different runs of the algorithm on the same sample using random initial configurations and the corresponding error bars (standard deviation) are shown at representative points.

If the dynamics is now happening on a two dimensional surface, the nucleation rate 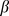 will have units of 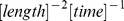 and by dimensional analysis it is predicted from the one-dimensional analysis that the time to reach a given coverage 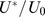 (where 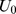 and 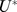 now denote initial and final areas in units of 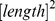) is given by
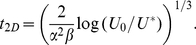
(4)Similarly, the total area is expected to evolve according to an equation on the form
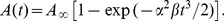
(5)We have tested the forms of Eqs. (4) and (5) by numerical simulations (see Figs. 3 and 4) using two versions of an off-lattice growth model. In both simulations, it has been assumed that new lichens nucleate in the unoccupied space 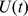 at a rate 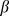 such that the expected number of lichens introduced at any given time is 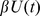. In one simulation the lichens expand uniformly from their point of nucleation with velocities 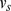 which are species-dependent. Locally, the growth continues in the unoccupied space and stops when another lichen boundary is met. The other simulation is inspired by an off-lattice Eden growth model [13]. Here a lichen consists of a number of particles that multiply at a rate 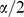. The particle production occurs only in the empty space, i.e. new particles will not overlap with preexisting particles and the growth will be limited to the perimeter of the lichens. More details on the algorithm is provided in the Materials and Methods section.

### Predicting the dynamical history

**Figure 5:**
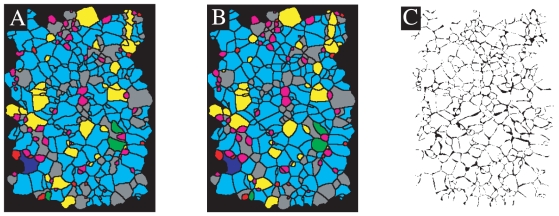
Quality of the prediction of the dynamical history. Panel A), synthetic image of a part of the pattern in Fig. 1B 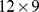 cm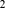. Panel B), the model prediction of pattern A). Panel C), comparison between prediction and observation.

Fig. 1B shows a rock covered with a community of 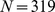 lichens and 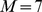 species. The species have been identified and are in Fig. 5A marked by synthetic colors. The corresponding names are given in Table 1. We now introduce a technique for estimating the dynamical history of a general community.

**Table 1:** List of species presented in Fig. 5A and corresponding growth velocities relative to the fastest growing species.

Species	Color	Relative velocity
*Bellemerea alpina* (Sommerf.) Clauzade & Roux	yellow	0.80
*Bellemerea cinereorufescens* (Ach.) Clauzade & Roux	cyan	1.00
*Rhizocarpon geographicum* (L.) DC.	purple	0.55
*Rhizocarpon polycarpum* (Hepp) Th. Fr.	grey	0.98
*Lecanora polytropa* (Hoffm.) Rabenh.	red	0.63
*Lecanora intricata* (Ach.) Ach.	green	0.71
*Aspicilia* sp.	blue	0.86

In the Monte Carlo simulations the velocities are always computed relative to the fastest growing species and therefore in order to achieve the real velocities, we must calibrate the values in the table with a known velocity of at least one species.

The final pattern of a community is determined by the nucleation points (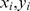), nucleation times, 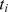, and growth rates, 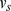, of different lichen species, where the subscript 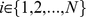 runs over the number 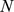 of individuals and 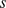 over the number 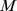 of species 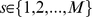. For simplicity, we shall neglect the influence of variations in the local environment and assume that the growth rates only vary from species to species and not within one species. For realistic population sizes of the lichens it quickly becomes intractable to determine the initial conditions by brute force and we shall therefore implement a Monte Carlo method. The error of our estimate of the initial conditions is calculated by summing the mismatch in coverage of individual lichens 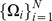 computed by Monte Carlo simulations and the original coverage 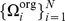. In our algorithm 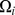 is given by the set of pixels that constitute a fully developed individual. Our method is implemented as follows, first we seed the algorithm by a guess of the initial condition. From the seed data, we then generate the corresponding lichen pattern and compare it with the real pattern. The mismatch in coverage of the individual lichens is computed and used to update the estimate of the initial conditions. During the update, noise is added to the estimate in order for the algorithm not to get stuck in a suboptimal configuration. Finally, with the updated estimate the algorithm starts over again.

**Figure 6:**
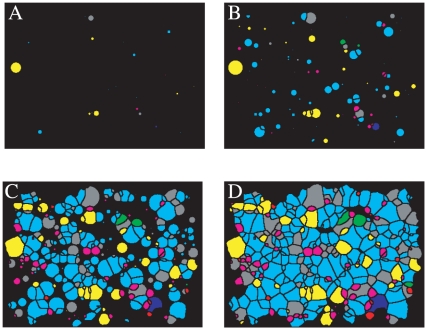
Snapshots of the temporal evolution of a lichen community. The evolution is estimated from applying the inverse method outlined in the text to a real sample of lichens. The snapshots are taken at A) 30%, B) 40%, C) 60% and D) 80% of the time needed for total coverage.

The nucleation sites, nucleation times and growth rates of individual lichens in a natural sample e.g. the one shown in Fig. 5A can be estimated using the aforementioned Monte Carlo method. The growth velocities are estimated relative to the fastest growing species in the community and therefore to get the real velocities the predictions have to be scaled with a known velocity for one of the species. One way to find real velocities is to identify species on partially covered rocks such as the one shown in Fig. 1B. Here we take a typical velocity to be approximately 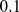 mm/year (consistent with growth rates reported in [8]) and thus arrive at an estimated coverage time of 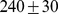 (s.d) years for the sample shown in Fig. 5A. In Fig. 5B we have used the estimated parameters for the individual lichens to run the forward dynamics. The difference between the model prediction and the real pattern is shown in Fig. 5C, where the black and white regions are regions where the predicted coverage is erroneous respectively correct. The mismatch is approximately 10% and can to a large extent be accounted for by the natural fluctuations in the contact boundaries which our method cannot resolve. Four stages of the temporal evolution leading to Fig. 5B are shown in Fig. 6. It is observed that initially almost none of the adjacent individuals are in contact and only at times when most of the lichens are nucleated do we start to see contours of the final boundary network. In Fig. 4 the predicted coverage in time of the real sample is compared to the model prediction, Eq. (5).

Even though our model only has two parameters and one parameter is fixed by the average velocity, we find that it fits the temporal evolution of the area coverage very well. Using the coverage time computed by the Monte Carlo simulation, we are able to estimate the nucleation rate 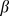 by Eq. (5), i.e. for the sample considered here, we find that historically there has been approximately 50–100 new lichens forming per 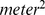 per 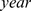.

## Discussion

In general, the spatial evolution of crustose lichen communities is controlled by species specific growth rates and a rate for how often new lichens are established in unoccupied areas. We find that on the scale of 10 cm an isotropic nucleation rate provides a very good fit to field samples. In other words, the establishment of new lichens is not dominated by spores dispersed by nearest neighbors. In general, dispersal mechanisms such as wind and water flows operate over long distances and can account for floristic affinities between even remote landmasses [14].

Although crustose lichens, if undisturbed in their growth, cover areas proportional to the square of their age, we find that the uncovered area retracts super-exponentially in time due to the establishment of new lichens. In lichenometric dating, the age of a community is usually estimated by measuring the diameters of individual lichens [15] or doing statistics over multiple lichens [16], however, in densely populated communities this will systematically underestimate the age by not taking into account the formation of stationary contact boundaries. In fact, we show that the time needed to reach a given coverage is a non-trivial function of both the vegetative propagation and the establishment of new lichens. If there were no nucleation, the coverage time would simply be inversely proportional to the growth velocity. That being said, the competitive advantage of being able to nucleate in remote areas is clearly emphasized by the resulting acceleration, predicted by Eq. (4), of coverage with time.

## Materials and Methods

In the Monte Carlo method, the spatial configuration of a given lichen community is computed from the estimated nucleation points (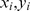), nucleation times, 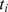, and growth rates, 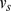 of the individual lichens in the following way. First we create a map using a square lattice with a resolution identical to that of the image of the lichen community. Then the evolution of the community is followed locally by letting each point on the map represent the shortest time it takes for lichens nearby to reach that point. We only track the time needed to reach points which are less than 5 lattice units away from existing lichens (in that way, we follow a method similar to the principles of Huygens wave propagation, where the lichen front represents the wave front and the distance map represents the secondary point waves). We update the evolution of the lichens iteratively, with the condition that the front cannot move more than one lattice unit in one iteration. When the local front of a lichen passes an unoccupied lattice point it is marked as part of the lichen. Other lichens reaching the same lattice point, but at a later time, will not change the state of the grid point, and the lichen front advancement in that direction is halted. The nucleation of new lichens at later stages in the evolution is simply represented by a local marking of a lattice point.

In addition to the algorithm used in the Monte Carlo simulation, we have implemented an off-lattice stochastic computer model inspired by the Eden growth model. In this model, lichens are made up of small non-overlapping spherical particles with a radius 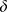. At time 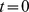, we place a seed particle at a point in space (where space here refers to a square of unit size and with periodic boundary conditions). New particles are then introduced at random points in the neighborhood of the seed particle (at at distance 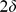) and at a given rate (equivalent to the growth velocity). The introduced particles all act as new seed particles and multiply with the same rate as the mother particle. A particle can only multiply if there is an empty site next to it that is, a new particle does not overlap with any of the existing particles. In addition to the particle production, we also nucleate new seed particles with a rate 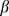 at random positions in space. The family of particles growing from different nucleation sites are marked with different colors and may have different multiplication rates. If a nucleation site is chosen on top of existing particles it is disregarded, i.e. the average number of nucleation events in a time step is proportional to the unoccupied area. Locally we construct a map of nearby particles, such that when new particles are introduced, we only have to check for available space between local particles. The data in Fig. 3 were generated using 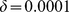.
